# Best practice and needs for improvement in the chain of care for persons with dementia in Sweden: a qualitative study based on focus group interviews

**DOI:** 10.1186/s12913-014-0596-z

**Published:** 2014-11-30

**Authors:** Christina Bökberg, Gerd Ahlström, Staffan Karlsson, Ingalill Rahm Hallberg, Ann-Christin Janlöv

**Affiliations:** Department of Health Sciences, Faculty of Medicine, Lund University, P.O. Box 157, SE–221 00 Lund, Sweden; School of Health and Society, Kristianstad University, SE–291 88 Kristianstad, Sweden

**Keywords:** Dementia care, Best practice, Transitions, Health care, Chain of care, Home care, Nursing home, Professional care providers, Nursing

## Abstract

**Background:**

Persons with dementia receive health care and social services from a wide range of professional care providers during the disease trajectory, presenting risks of miscommunication, duplication and/or missed nursing interventions. Accordingly, the aim of this study was to investigate professional care providers’ views on conditions for best practice in terms of collaboration and improvement needs in the chain of care from early to end-of-life stage for persons with dementia in Sweden.

**Methods:**

The study had a qualitative design based on three focus group interviews. A strategic sample of 23 professional care providers was included. Data were subjected to content analysis based on the three stages of dementia (early, moderate, end-of-life).

**Results:**

The results were divided into five categories: *Diagnosis is a prerequisite for specialized dementia care, Creating routines in the chain of care, Competent staff a prerequisite for high-quality care, Day care facilitates transition in the chain of care and Next-of-kin participation is a prerequisite for continuity in the chain of care.* It was clear that, according to the participants, best practice in dementia care in Sweden is not achieved in every respect. It appeared that transitions of care between different organizations are critical events which need to be improved. The further the disease progresses, the less collaboration there seems to be among professional care providers, which is when the next of kin are usually called upon to maintain continuity in the chain of care.

**Conclusions:**

The results indicate that, according to the care providers, best practice in terms of collaboration is achieved to a higher degree during the early stage of dementia compared with the moderate and end-of-life stages. Lack of best practice strategies during these stages makes it difficult to meet the needs of persons with dementia and reduce the burden for next of kin. These are experiences to be taken into account to improve the quality of dementia care. Implementation research is needed to develop strategies for best practice on the basis of national knowledge-based guidelines and to apply these strategies in the moderate and end-of-life stages.

## Background

The global increase in the number of older people and in the number of people afflicted with dementia [1,2] has implications for the organization and provision of health care and social services [3]. Dementia implies progressive loss of cognitive and functional abilities over a series of predictable stages or phases, causing increased dependency on others in managing the daily life [4,5]. Persons with dementia receive more health care in general than do persons without dementia, from a wide range of professional care providers employed by municipalities and county councils across acute care and nursing home settings [4]. This presents risks of miscommunication, duplication and/or missed nursing interventions [6]. During the disease progress, a competent nurse can smooth the way for the patient by identifying needs and problems and offering support, guidance and planning [7]. Therefore, to reflect on experiences of professional care providers regarding best practice strategies is one way to improve dementia care.

The trend in Sweden is that an increasing proportion of the elderly population receives health care and social services at home [8-10] with support from next of kin [10]. Although the sense of identity and integrity of the person with dementia is promoted by living in the own home, the burden increases for their next of kin as the disease progresses [3]. In Sweden it is voluntary for next of kin to care for elderly people. Still, next of kin remain the cornerstone in the care of persons with dementia, although their own need for support is often overlooked [11]. One way to meet the needs of persons with dementia and lower the burden on next of kin is by pursuing best practice strategies. Best practice can be defined as a technique or methodology that, “*through experience and research, has given evidence of being reliable and of leading to a desired result. […]Best practice in any field is a commitment using all the knowledge and technology at one’s disposal to ensure success*” [12] [*quote* p 15]. The Swedish National Guidelines for Care in Cases of Dementia [13] include recommendations concerning support to next of kin, multi-professional teamwork and person-centred care. In dementia care, person-centred care has been synonymous with best practice [14]. There is no consensus regarding the concept of person-centred care, but Edvardsson et al. [15], have indicated that [*quote* p 2614] “*the core aspect is* “*promoting a continuation of self and normality*”. Person-centred care highlights the importance of knowing the person behind the patient and involves interaction between professional care providers, the person with dementia and their next of kin according to each individual’s needs, personality and abilities [15].

The Swedish care system consists of two main bodies and two different legislations with divided responsibility between them. The county councils are responsible for the investigation leading to dementia diagnosis, treatment and follow-up. The municipalities are responsible for health care and social service, providing assistance for those older persons receiving day care, care at home or living in a nursing home [16,17]. The Swedish legislation puts emphasis on access to health services based on assessment of individual needs and availability to all members of society on equal terms [18,19]. During the last decade, there has been an increase in numbers of private care providers in primary care and care of the elderly. Moreover, politicians have promoted free choice of primary health care, which tends to fragment and have adverse effects on integrated care [20,21]. Person-centred care, i.e. best practice, is difficult to achieve in a fragmented care system. This situation has created the need for increased continuity and improved collaboration among professional care providers involved in the chain of care for persons with dementia.

Given this complexity, it is important to investigate how transitions in the chain of care are coordinated, since fragmentation of responsibilities may result in information loss and discontinuity, leading to unmet care needs for persons with dementia [22,23]. The experiences and perspectives of professional care providers regarding best dementia practice can contribute to the development of dementia care that can meet the needs of persons with dementia and their next of kin.

### Aim

The aim of this study was to investigate professional care providers’ views on conditions for best practice in terms of collaboration and improvement needs in the chain of care from early to end-of-life stage for persons with dementia in Sweden.

## Methods

### Design

This study had a qualitative design using focus group interviews. The study was part of the European research project RightTimePlaceCare (RTPC) aiming to improve care and services for European citizens with dementia through development of strategies for best practice, from diagnosis to end-of-life care. The countries participating in the project were England, Estonia, Finland, France, Germany, the Netherlands, Spain and Sweden [24]. This study was approved by the Regional Ethics Review Board in Lund, Sweden (reference number: 2010/538).

### Participants

A strategic sampling of professional care providers and workplaces was made to obtain variation in experiences and perspectives on best practice in terms of collaboration and improvement needs in the chain of care. The recruitment of participants was done through seven registered nurses specializing in dementia care, in four municipalities in both urban and rural areas in southern Sweden. These nurses gave oral information about the study to various professional care providers working in different types of care settings, representing memory clinics, home care, day care and nursing homes, all providing care for persons in different stages of the disease progress. After giving consent, the care providers were contacted by a researcher in the research team who gave them further information about the study process.

A total of 23 participants were included in the study. The participants were 28–61 years of age (mean age 52 years). Their work experience ranged between 3 and 42 years (mean 30 years) and all but one of those taking part were women. Each focus group consisted of a mixture of registered nurses with or without specialized education in dementia care, assistant nurses, occupational therapists and social workers. None of the physiotherapists who were approached consented to participate. The care organizations representing the chain of care were county councils [health care] and municipalities (health care and social service) [Table 1].

**Table 1:** **Descriptions of the participants in the three focus groups**

	**Focus group 1**	**Focus group 2**	**Focus group 3**
**County council**			
**Memory clinic**	Registered nurse specialized in dementia care		Occupational therapist
	Assistant nurse		
**Municipality**			
**Overall function**	Registered nurse specializing in dementia care	Registered nurse specializing in dementia care	Registered nurse specializing in dementia care
	Social worker	Social worker	Social worker
**Home care**	Occupational therapist	Occupational therapist	Occupational therapist
			Assistant nurse
	Assistant nurse		
**Day care**	Assistant nurse	Assistant nurse	Assistant nurse
**Nursing home**	Registered nurse	Registered nurse	Registered nurse
	Assistant nurse	Assistant nurse	Assistant nurse

### Focus group interviews

Focus group interviews were selected as data collection method as they were hoped to provide the opportunity to obtain professional care providers’ views and perceptions [25] regarding conditions for best practice. An interview guide was developed covering questions about their experiences of collaboration, information and channels of communication that work well in the chain of dementia care. Furthermore, follow-up questions concerning any deficiencies in the chain of care were included. Finally, the interview guide included questions asking the participants to describe their visions of best practice and make suggestions of improvement.

The 23 participants were divided into three groups of six to nine participants each. A focus group interview was conducted with each of these groups. Just before the interviews the participants were informed about the study both orally and in writing and were given the opportunity to ask questions before signing to confirm their informed consent. Each focus group interview was led by two registered nurses: a moderator (ACJ) and an observer (CB). The moderator is a researcher in the field of geriatric care and is experienced in conducting focus group interviews. The observer was a doctoral student with work experience in different areas of the health care system, including the nursing of persons with dementia.

All interviews followed a similar structure. The interview started with the moderator’s clarifying its purpose, and then posing questions based on the interview guide. The participants were encouraged to bring their views into the open and the observer helped the moderator in ensuring that the participants kept to the questions. The interviews were digitally recorded and then transcribed verbatim. Each interview took about 2 hours. Two of the interviews took place in a conference room at a university and one in a conference room in a nursing home. Data from the interviews were processed in such a manner that there should be no unauthorized access to them, and the results have been reported in such a manner as to maintain confidentiality.

### Analysis of the transcribed interviews

The text was analysed as a conversation, with the participants responding to each other as a group [25]. The interview texts were subjected to qualitative content analysis [26], performed by CB, and based on the stages of dementia (early, moderate, end-of-life). Firstly in this analysis, the transcripts were read through a number of times to get a sense of the whole. Secondly, expressions (sentences/paragraphs) of relevance to the study aim were identified and divided into meaning units. Thirdly, the meaning units were condensed at a descriptive level, keeping close to the text. Fourthly, the condensed meaning units were abstracted and labelled with a code. The interviews in their entirety served as a point of reference throughout the analytical process, in particular when deeper understanding was needed in respect of the meaning units and codes. Fifthly, the codes were thoroughly compared regarding similarities and differences, before categories were created [Table 2]. Three of the authors (GA, SK and ACJ) separately read and critically reviewed the meaning units, codes and categories in relation to the interview texts, reflected on them and then discussed them with the other authors in several combined meetings. This procedure made it possible to uncover as many qualities as possible within the text and reach a consensus concerning the results.

**Table 2:** **Example of analysis steps**

**Text**	**Meaning unit**	**Meaning units condensed at the descriptive level**	**Meaning unit abstracted and labelled with a code**	**Category**
Assistant nurse, nursing home: “Mm, it is that way, too. I imagine it’s a frightening disease	Participants commented that it must be a frightening disease to get – to receive a fatal diagnosis, find out that you will continuously deteriorate and become dependent on other people – and that this entails a psychological defence mechanism (which prevents the person from seeking help)	Knowledge about the development of the dementia disease is frightening, and may be why a person fails to seek help	Knowledge makes the person afraid of seeking a diagnosis and help.	Diagnosis is a prerequisite for specialized dementia care
Dementia nurse: “That’s the thing …”				
Assistant nurse, nursing home: “It’s a horrible disease to get. I mean, to get a fatal diagnosis – that’s what it is … plus, the fact that you’re going to be continuously deteriorating … and won’t be able to … and you’ll be dependent on other people … and what kind of people are you going to end up being dependent on?				
Dementia nurse: “It’s a psychological defence mechanism, too.”				
Many: “Yeah …”				
Assistant nurse, nursing home: “It’s completely natural …”				

### Results

The analysis of the text revealed five categories concerning the participants’ views on best practice and needs for improvement in the chain of care from early to end-of-life care for persons with dementia: *Diagnosis is a prerequisite for specialized dementia care, Creating routines in the chain of care, Competent staff a prerequisite for high-quality care, Day care facilitates transition in the chain of care* and *Next-of-kin participation is a prerequisite for continuity in the chain of care.* Reported results are presented according to the stages of dementia.

### Diagnosis is a prerequisite for specialized dementia care

The participants emphasized that best practice in dementia care starts with the diagnosis, enabling individualization of care. Therefore, when professional care providers identify early signs of dementia, i.e. cognitive impairment, they try to see to it that the person gets a diagnosis. This involves contacting general practitioners (GPs), giving information and asking for examinations. Where the GP has no time or expertise, such examinations are not performed, and instead, the person gets treatment in the form of symptom relief with psychotropic drugs. The participants discussed barriers for people seeking help and a diagnosis. Lack of knowledge and remaining social stigmas regarding dementia, but also desire to manage self-care and efforts by next of kin to compensate for decreased abilities were reported as barriers.

Regarding later in the disease trajectory, the participants said that lack of a diagnosis had consequences for the person with dementia, as it hindered the provision of care and the prescription of assistive technology for them. The participants described their feelings of powerless in these situations and discussed concerns that such persons do not receive high-quality (or even adequate) care.

Concerning the end-of-life stage, only persons with a dementia diagnosis have access to nursing homes specializing in dementia care. However, often dementia patients are stigmatized for living in such a nursing home. The participants were also concerned that it could be depressing for persons with dementia to move to this kind of nursing home too early and find themselves living close to persons with more advanced dementia. In light of this, the participants suggested that persons with milder cognitive impairment may be better off in ordinary nursing homes. The participants also emphasized that dementia care should be based on the individual person’s needs and wishes, instead of treating people living in nursing homes like a group.

**Social worker:***Nah… — if you’ve got the diagnosis and you’ve got the need, it’s special dementia housing that’s the answer.*

**Dementia nurse:***You can see people who haven’t got a dementia diagnosis and are in ordinary special housing — and that can be difficult, of course. In some places it works just fine, but in others there are problems, for sure.*

**Dementia nurse:***I could cite several examples of people who’ve sunk into pretty deep depression … . They’re lucid and capable enough in spite of their dementia but they’ve landed among people who’ve gone a long way downhill and whose behaviour is troublesome. Ordinary housing would have been alright for them.*

**Social worker:***Ordinary special housing.*

**Dementia nurse:***Ordinary special housing — and that makes me wonder … there aren’t many there who can say, “I’ve got dementia.”*

**Several participants:***Nah… nah…*

**Dementia nurse:***But naturally the basic idea is “dementia diagnosis at special dementia housing”.*

**Dementia nurse:***If you’re going to consider what can be worse there it’s definitely individual thinking. There’s far too much group thinking.*

**Social worker:***Yes, what it comes down to is interest in the individual.*

**Moderator:***Which is to say, making the most of every individual’s resources …* [Interview 2]

### Creating routines in the chain of care

Best practice concerning collaboration was described by the participants as calling for routines for information exchange during transitions in the chain of care, and having shared goals for the care. It was suggested that to facilitate collaboration between care organizations, a special coordinator is required. In the early stage, registered nurses working in primary care who have special training in dementia care often bridge the gap between specialist care in the hospitals and elderly care in the municipalities. These nurses normally use a special form to facilitate information exchange and planning for future care needs, the participants said.

Concerning the moderate dementia stage, the participants expressed the view that this is where routines for information exchange begin to fail. Routines mentioned as being in need of improvement are both those needed to facilitate transition between care organizations and those required between professional care providers. The participants emphasized that flaws in routines for information exchange occur between county councils and municipalities, during transitions from one type of care to another (hospital care, short-term care, nursing home care and home care) and in respect of written information between professional care providers in home care. The participants also reported that written information exchange is hindered by the Swedish secrecy law concerning social services and health care.

Regarding the end-of-life stage, the participants expressed concern that there are gaps in collaboration during transition between home care and the nursing home; further, they said that the routines for information exchange need to be improved to guarantee good and secure care. They explained that in some cases, there needs to be a quick decision about this transition, making it difficult to find time for information exchange. The professional care providers taking over might then lack the requisite information about the person’s needs and about the interventions that are needed.

**Occupational therapist:***The fact is, we don’t always get the information — but then suddenly an intervention’s needed and of course that’s when you need to know as much as possible about the person. There’s got to be an improvement.*

**Assistant nurse, nursing home:***It all just happens very quickly. The room can be vacated one day and occupied again the next. We hardly know ourselves that somebody new is going to be there.* [Interview 2]

### Competent staff a prerequisite for high-quality care

The participants stated that best practice, in all stages, is when professional care providers have the requisite knowledge about the person and the dementia trajectory to facilitate transitions in the chain of care. A well-functioning collaboration between knowledgeable and committed professional care providers was seen by the participants as implying fewer hospital visits for persons with dementia. With regard to the early stage, they emphasized medical competence, based on the importance of getting a dementia diagnosis.

Discussing the moderate stage, the participants indicated that professional care providers working in home care often lack special education about dementia; also, they are forced to work under time pressure and stress. The participants also mentioned that persons with dementia sometimes behave in ways that may be considered improper. If the response to such behaviour is a sigh, for instance, or a rolling of the eyes (whether because of incompetence or because of time pressure), the person with dementia will be bewildered. To provide security and continuity in the care, it was proposed, there should be a special group in home care with both expertise in dementia and extra time for home visits.

**Assistant nurse, home care:***In the first place there should be continuity … and there should be someone who knows about dementia. I mean, it’s not like going to the home of …*

**Registered nurse:***… just anybody.*

**Assistant nurse, home care:***… of somebody who has just aged normally, you might say. I mean, it calls for something altogether different. Patience and time and …*

**Several participants:***Mm … mm … mm …*

**Occupational therapist:***And when it comes to dementia there’s that important bit … — it’s just like you say, that … It’s not everybody can cope with the job when dementia’s involved.* [Interview 3]

Concerning the end-of-life stage of dementia, the participants said that a specific competence is required and staff needs training to be able to correctly interpret symptoms and behaviour. The participants discussed that symptoms like pain can be expressed in a different way by persons with dementia compared with those not suffering from the disease. When professional care providers lack the competence to interpret such symptoms correctly persons with dementia will not have their needs fulfilled. The participants did, however, add that misinterpretation of symptoms is common in the end-of-life stage.

**Dementia nurse:***I think it’s important that even when the disease is far advanced there’s respect for the person and you have the right knowledge about pain and other … — well, other purely bodily symptoms. About how they can be expressed when it comes to these people … — so that they can get the same care as other people. You see, I don’t think it’s really …*

**Registered nurse:***Nah …*

**Dementia nurse:***… quite that way today. No, it isn’t.*

**Registered nurse:***Of course a lot of people with dementia show their pain in a different way than we do …*

**Assistant nurse:***Exactly.*

**Registered nurse:***… because they can’t really say what it is.*

**Dementia nurse:***Yes, and often there’s not the education … . You’re working directly with the sick person but you don’t always have the education you ought to have.* [Interview 3]

### Day care facilitates transition in the chain of care

The participants considered visiting day care makes it easier for persons with dementia and their next of kin to establish relations with professional care providers. They further indicated that transitions in the chain of care become smoother when access to day care is given, enabling a gradual increase in day care, nursing interventions and support. This gradual increase in support makes it possible for the person with dementia to stay longer in their own home if they wish to. In the early stage, when planning for care and putting together the person’s life story, professional care providers talk to the person with dementia and their next of kin individually. Thus a good relationship is established which makes it easier for the person with dementia and their next of kin to ask for more help or other care alternatives later in the disease trajectory. The participants also said that during day care, social contacts increase, making it possible for the person with dementia to share experiences of the illness (and share tips about how to deal with everyday life) with other persons in the same situation. Thus it is important, the participants emphasized, that the person with dementia should begin with day care in the early stage.

Moving on to the moderate stage, the participants described the person with dementia as being in a borderland between home care and nursing home care, and said that the best type of care can be difficult to find. Day care was described as best practice for facilitating transition in this part of the chain of care in that day care constitutes a link between home care and nursing home care. Professional care providers working in home care and nursing homes are often invited to go to the day care centre and establish contact with the person with dementia. In that way, said the participants, day care increases the person’s sense of security and makes it easier for them, as well as gives them greater motivation, to move into a nursing home in the end-of-life stage.

**Assistant nurse, home care:***When I meet them they’re somewhere in a … grey zone. And it’s not always clear what they really need. Whether they’re going to go on living at home or not. I mean, they haven’t reached the stage yet where they can see themselves moving. Neither the next of kin nor we ourselves feel altogether comfortable about their going on living at home, so it’s a sort of borderland … . You don’t really quite know what’s best. And how much of a danger are they living at home — to themselves and … ? So they’re neither at the beginning nor at the end. It seems to me it’s a difficult phase.*

**Assistant nurse, day care:***So then it can be good, of course, to be there [at the day care centre], whether it’s one day a week or five days a week. Because then both the person with dementia and the next of kin get to know us.*[Interview 3]

### Next-of-kin participation is a prerequisite for continuity in the chain of care

The participants stressed that it is important to create a good relationship with the next of kin already in the early stage. This relationship was considered important, on the one hand because it facilitates the professional care providers receiving information from the next of kin; on the other, because the next of kin receive information, support and help as the disease progresses.

Where the moderate stage is concerned, the participants emphasized the importance of involving both the person with dementia and the next of kin when discussing care and care alternatives, as it is important that the person should receive care in line with expressed needs and wishes.

In the end-of-life stage, when it becomes necessary for the person with dementia to move to a nursing home, the next of kin are an important source of information, said the participants. However, the decision as to this move can be connected with guilt for next of kin, so they actively seek information from the professional care providers about different nursing homes in order to find best care possible. The participants emphasized that although next of kin seek and receive information about nursing homes their knowledge about these homes generally remains vague. The participants spoke of a change of mind-set on the part of the next of kin when the dementia sufferer is transferred to care in a nursing home: often they are calm at first and exhibit a sense of security, but after a while they start wondering whether other types of care may have worked better.

**Registered nurse:***I meet the next of kin after the move’s been made — a month, two months, three …*

**Registered nurse:***At first they’re very pleased and think … — no, they can* see *that Mum or Dad’s being looked after. There’s a sense of security.*

**Dementia nurse:***They can relax.*

**Several participants:***Yes, mm …*

**Registered nurse:***But then, after a bit of time has passed and things aren’t always 100% at the nursing home, they start thinking that, well, perhaps there was a better solution …*

**Dementia nurse:***I think it’s a question of information, communication and collaboration.* [Interview 1]

The participants emphasized how particularly important it is that during the end-of-life stage, if the person with dementia is too ill to express any wishes concerning care and has not previously done so, professional care providers should communicate and collaborate with the next of kin in care planning.

## Discussion

The results show that the health care and social service system do not achieve best practice in terms of collaboration in every respect and those certain critical events, i.e. transitions in the chain of care, are in need of improvement. The first critical transition is illustrated in the category *Diagnosis is a prerequisite for specialized dementia care.* Lack of a diagnosis has consequences for the person with dementia in that they are then denied access to specialized dementia care (dementia teams, day care and nursing homes specializing in dementia). Yet in spite of this, not every person with cognitive impairment undergoes medical examination and gets a diagnosis. Moise and colleagues [4] state that the dementia diagnosis is important in enabling the person with dementia and their next of kin to plan for the future, to understand the illness and to make informed decisions. Persons with dementia should also be encouraged to take part in decision making for as long as possible to maintain their dignity and self-esteem [27]. The participants stressed that an early diagnosis, made when it can still be understood by the patient, benefits both the person with dementia and the next of kin. They can at this stage have an influence with regard to care alternatives and access to treatments which may delay cognitive decline and can more readily participate in planning for the future [4,28]. The participants added that avoidance of the patient’s cognitive problems, because of remaining prejudices and social stigma regarding dementia is a barrier to diagnosis. Batsch & Mittelman [27] found that persons diagnosed as having dementia reported losing former friends but at the same time establishing new relationships with persons in the same situation. However, the negative social perception of dementia leads to greater isolation. Rose & Palan Lopez [29] proposed that nurses should reduce the stigma of dementia through open, honest discussion and by empowering the person with dementia and their next of kin in their new roles. As early diagnosis implies obtaining access to specialized dementia care, there is an urgent need to take action to dispel remaining prejudices about dementia and any stigma associated with it.

When the disease progresses from the early to the moderate stage, there is an increase in the need for support in activities of daily life. Day care plays an important role when the need for nursing intervention and support increase, and it postpones the move to a nursing home, as described under *Day care facilitates transition in the chain of care*. When the disease progresses further, day care facilitates the change from home care to the nursing home by giving the person with dementia and their next of kin the opportunity to establish contacts with professional care providers who will thereafter make them feel comfortable about asking for more help or other care alternatives. Day care is based on the principle of providing the elderly with the requisite support for achieving better quality of life and maintaining independence for as long as possible [18,19]. Day care was described by the participants as providing best practice during transitions throughout the chain of care, making transitions smoother. Day care gives the person with dementia a better chance to go on living at home in spite of a gradually increasing need for support.

The results revealed that there is a need for improvement with regard to certain critical events in the disease trajectory. In the early stage, professional care providers collaborate with each other and with the person with dementia and their next of kin, in accordance with best practice. However, in the moderate and end-of-life stages, when the disease has progressed and the patient’s ability to express their needs and wishes has diminished, the collaboration can break down: the less specialized in dementia care the professional care providers, the less collaboration there tends to be. The category *Creating routines in the chain of care* brought out the fact that the transition from the own home to a nursing home can sometimes be based on a sudden decision, which combined with a lack of information routines can mean that the needs of the person with dementia are not properly met. Negative consequences for the person with dementia moving into a nursing home can be reduced by properly preparing the transition, exchanging information and smoothing the path for visits in accordance with the Swedish National Guidelines for Care in Cases of Dementia [13]. The category *Competent staff a prerequisite for high-quality care* revealed that in the early stage, although next of kin can compensate for the person with dementia’s decreased abilities, many professional care providers lack the requisite competence and knowledge to determine what help is needed. At the end-of-life stage, when the person with dementia is heavily dependent on others, many professional care providers lack requisite special knowledge concerning dementia, often making it impossible for them to correctly interpret symptoms and offer adequate care and treatment. Similar trends were found in a newly published study [30] reporting that while in the early stage, professional care providers with at least a Bachelor’s degree were often involved, in later stages the care was provided by professional care providers with a lower level of formal nursing education or none at all.

The lack of best practice strategies, i.e. flaws in routines for collaboration and information exchange in the moderate and end-of-life stages, has consequences for the person with dementia as well as for their next of kin because no one has a holistic perspective on the care. Other Swedish researchers have reported difficulties in collaboration in the chain of care for persons with chronic diseases [21], supporting the results in this study. When the collaboration among professional care providers fails, there is an increase in participation in the care by next of kin as information couriers. This is shown in the category *Next-of-kin participation is a prerequisite for continuity in the chain of care*. Transferred responsibility of care to the next of kin regarding continuity in the chain of care may increase their burden and putting expectations on them. Swedish municipalities are legally obligated to provide support to next of kin caring for elderly people [18] but what the law in fact has given the next of kin is an assessment of their needs but no services [31]. This situation also raises questions about situations where next of kin are absent. Then professional care providers need to take responsibility for advocacy for persons with dementia to ensure continuity in the chain of care. A previous study has shown that case managers could help persons with dementia to navigate through the health care system in terms of contacting and interacting with professional care providers [32]. However, case managers are not yet common in the Swedish dementia care system. Improvement of best practice to meet the needs of persons with dementia and their next of kin and better collaboration between professional care providers is therefore urgently needed.

As discussed, when the disease progresses the responsibilities of next of kin increases [4]. Having to perform an increasing amount of care and service may have a negative impact on the wellbeing of next of kin. There seems to be a gender difference in next of kin providing dementia care. For instance, adult daughters and daughters-in-law are more likely than sons and sons-in-law to provide routine assistance with household chores and personal care, and also spent more hours per week in providing other assistance needs [33]. Therefore there is a need in changes in health care policies to fully recognize and support the role of next of kin as a health care resource. The support and information need to be adjusted in accordance with the stage of dementia and the relationship of next of kin with the person with dementia (e.g. spouse or child). In a Dutch study, 67% of the next of kin caring for a person with dementia at home with support from professional care providers reported that they needed further assistance. This need differed depending on the relation to the person with dementia. Spouses asked for relief and emotional support from professional care providers to deal with anger, fear and anxiety, while children of persons with dementia asked for more information about the disease process and better co-ordination of the professional care [34]. Other studies have shown that multi-component interventions, such as psychosocial interventions combined with educational programmes and support for next of kin, seem to be the most effective [35,36]. The present study reveals that in Sweden, the collaboration and information exchange during progress from moderate to end-of-life dementia stage has flaws which need to be remedied to guarantee good and secure care, and avoid increasing the burden on next of kin.

The results indicate that to establish a trusting relationship, professional care providers’ collaboration in the chain of care during the disease process should focus more on the person with dementia and their next of kin. In conclusion, an individual adaptation of care and services to the person with dementia and their next of kin, and proactive care throughout the disease trajectory is regarded as best practice. Such approach indicates compliance with the concept of person-centred care.

### Methodological considerations

To ensure trustworthiness, we evaluated the credibility, dependability and transferability of the procedures used to generate valid results in this qualitative study [26]. Even though the study was designed to obtain trustworthy results, certain aspects need to be reflected on when interpreting the results and transferring them to other settings. To strengthen dependability, all three focus group interviews were conducted within 5 months and by the same researchers. Use of an interview guide ensured that all the interviews included the same overall questions. In order to meet the criterion of credibility a purposeful sample of types of care and of various professional care providers was obtained. All but one of the participants was female, reflecting the fact that nursing is a female-dominated occupation. Hence, the sample can be said to be representative of the professional care providers’ structure in the chain of care for persons with dementia. None of the physiotherapists invited to participate consented to do so. A reason for this may be that physiotherapists work more independently and are therefore not directly involved in the chain of care for persons with dementia.

Focus group interviews were chosen for the collection of data. One limitation of this is that the method gives only a picture of the group as a whole, providing the prevailing perceptions of the group, but not the exact number of people for and against this or that perception [25]. Another issue is that participants in focus group interviews do not always feel free to discuss sensitive and personal experiences and perceptions, especially if some of the other focus group participants are people they know and work with, as was the case here. This could limit the nature and amount of data obtained in the study. However, all participants took part in the discussions and spoke openly about the topics of interest, which indicates that they were confident with the interview situation. In the last interview no new information was obtained and no new insights were gained. To achieve trustworthiness, the authors independently analysed the material, but interpretations and conclusions were constantly checked and discussed until a consensus was reached regarding the codes and categories. To demonstrate trustworthiness of the categories, a thorough description of the analytical process and excerpts from the interviews are provided. To facilitate the decision as to whether the results are transferable to other contexts or not, descriptions of the context, the selection and characteristics of participants and the data collection and analytical process are given. A rich presentation, in this study, of the results together with appropriate quotations further enhances transferability.

## Conclusions

The main contribution to knowledge made by the present study is that best practice in terms of collaboration seems to be better achieved during care in the early stage of the dementia trajectory but is often not fully achieved during the moderate and end-of-life stages. Lack of best practice strategies during these stages makes it difficult to meet the needs of persons with dementia and reduce the burden on next of kin. These are experiences to be taken into account to enhance the quality of dementia care. Implementation research is needed to develop strategies for best practice from national knowledge-based guidelines and to apply these during collaboration in care during the moderate and end-of-life stages of dementia.
